# Clinical application of clustered-AChR for the detection of SNMG

**DOI:** 10.1038/srep10193

**Published:** 2015-06-11

**Authors:** Guang Zhao, Xiaoqing Wang, Xiaowen Yu, Xiutian Zhang, Yangtai Guan, Jianming Jiang

**Affiliations:** 1 Department of Neurology, Changhai Hospital, Second Military Medical University, Shanghai, China; 2 Department of Neurology, Renji Hospital, Shanghai Jiaotong University, Shanghai, China

## Abstract

Myasthenia gravis (MG) is an autoantibody-mediated disease of the neuromuscular junction (NMJ). However, accumulating evidence has indicated that MG patients whose serum anti-acetylcholine receptor (AChR) antibodies are not detectable (serumnegative MG; SNMG) in routine assays share similar clinical features with anti-AChR antibody-positive MG patients. We hypothesized that SNMG patients would have low-affinity antibodies to AChRs that would not be detectable using traditional methods but that might be detected by binding to AChR on the cell membrane, particularly if they were clustered at the high density observed at the NMJ. We expressed AChR subunits with the clustering protein rapsyn (an AChR-associated protein at the synapse) in human embryonic kidney (HEK) cells, and we tested the binding of the antibodies using immunofluorescence. With this approach, AChR antibodies to rapsyn-clustered AChR could be detected in the sera from 45.83% (11/24) of SNMG patients, as confirmed with fluorescence-activated cell sorting (FACS). This was the first application in China of cell-based AChR antibody detection. More importantly, this sensitive (and specific) approach could significantly increase the diagnosis rate of SNMG.

Myasthenia gravis (MG) is a relatively rare, but often severe disorder of neuromuscular transmission that causes considerable fatigue^1^,^2^. MG is initiated by immune reactions in the neuromuscular junction (NMJ)^3^. The majority of patients generate antibodies to the acetylcholine receptor (AChR), which is a pentamer composed of two α subunits and one of each of the other subunits: β, δ, and ε^4^. The second type of patient generates antibodies to muscle-specific tyrosine kinase (MuSK)^5^,^6^, and the third type of patient produces antibodies to lipoprotein receptor-related protein-4 (LRP4), which is an NMJ membrane protein that interacts with MuSK^7^. The proportion of patients with the latter two types of MG is very low, particularly in the Chinese population, and the primary autoantigen in Chinese patients with MG is to the AChR, which is clustered and anchored in the postsynaptic membrane of the NMJ by AChR-associated protein of the synapse (rapsyn)^8^.

Rapsyn is a 43-kDa postsynaptic tyrosine kinase receptor protein that is associated with AChRs at the NMJ and that plays an important role in the early stages of NMJ formation induced by nerve-released agrin^9^. *In vitro* studies have shown that rapsyn expressed at the cell surface forms clusters with AChR subunits^10^,^11^. Based on these findings, we sought to develop a cell-based assay to diagnose MG.

MG patients without AChR antibodies that can be detected using traditional methods are referred to as ‘seronegative’ MG (SNMG) patients; these individuals can have anti-AChR antibodies that bind only to high-density AChR clusters^12^. In particular, these AChR antibody-negative patients likely generate pathogenic antibodies that do not bind effectively to AChRs in solution but can bind strongly to *ex vivo* AChRs that are tightly aggregated at the cell surface^13^. Thus, we hypothesized that these SNMG patients would have low-affinity antibodies to AChRs that could not be detected using traditional methods, but that could be detected by binding to AChRs on the cell membrane, particularly if they were clustered at the high density observed at the NMJ. In the present study we tested this hypothesis by expressing recombinant AChR subunits with the clustering protein, rapsyn, in human embryonic kidney (HEK) cells, and we examined the antibody binding by immunofluorescence (as shown in the diagram, Fig. 1a).

Fifty-two MG patients were enrolled in this study from January 2013 to April 2014, including 24 who were diagnosed with SNMG and who were MuSK-negative based on traditional methods. Serum from these patients was further examined for the presence of anti-AChR antibodies using our novel cell-based assay.

## Materials and Methods

### Screening SNMG patients by ELISA

We collected serum from 52 patients, who were diagnosed with MG according to clinical and electromyographic criteria. We re-assayed all samples for anti-AChR and MuSK antibodies using ELISA (R&D, Inc., Minneapolis, MN, USA) according to the manufacturer’s protocol. We undertook our research in accordance with the relevant guidelines and laws in Shanghai: all the experimental protocols were approved by Changhai Hospital, and the ethics committee approval drafted by the regional government was waived for the blood samples because they were not obtained specifically for research purposes. All of the patients signed an informed consent form. Using ELISA, twenty-eight patients were positive for anti-AChR antibodies (AChR-MG), and 24 patients were defined as having SNMG based on the results obtained for healthy individuals in our laboratory using the same ELISA kit. These serum samples, as well as those from healthy subjects, used as negative controls, were subsequently examined using cell-based assays.

### Construction and transfection of AChR subunits

We transfected HEK 293 cells (provided by the Basic Medical School of Fudan University Biochemistry and Molecular Biology Laboratory) with plasmids containing each of the AChR subunits α, β, δ and ε, together with rapsyn-EGFP, as previously described^12^. The plasmids containing our target cDNA were generously provided by Biochemistry Shanghai Fudan University. We generated four groups of transfected cells: 1) cells transfected with empty vectors as the negative control; 2) cells transfected with GFP-RAPSYN only; 3) cells transfected with four subunits of the AChR without GFP-RAPSYN; and 4) cells transfected with all four AChR subunits and GFP-RAPSYN. The successful construction and expression of the transfected molecules were verified by western blotting.

### Cell-based assays

The transfected HEK293 cells described above were seeded in 24-well cell culture plates, and a coverslip was placed on the bottom of each well. After 24 hours, to ensure that the cells were adhering to the cover slips, the cells were fixed with 4% paraformaldehyde. Then, 15 μl of serum from each patient was added to a well to provide the primary antibody, and the reaction was allowed to occur for 1 hour at room temperature. After rinsing the wells with PBS 3 times, the secondary antibody (red) was added for 45 minutes at room temperature. Cells that reacted with serum AChR (red) and GFP were detected using confocal microscopy.

### FACS analysis

For flow cytometry analysis, 1 × 10^6^ cells/well were cultured in 6-well culture plates and transfected as described above. Approximately 48 hours later, the cells were detached from the plate using trypsin/EDTA and were centrifuged, counted and resuspended in cold PBS at a concentration of 10^6^ cells/ml. After washing, the cells were resuspended in 100 μl of binding buffer and were centrifuged, and the precipitate was incubated in 300 μl of 1% BSA (diluted at PBS) plus 15 μl of sera for 1 hour. After three washes, the cells were resuspended in 300 μl of Cy3-conjugated donkey anti-human IgG (Proteintech Group, USA) as the secondary antibody at a 1:500 dilution for 45 minutes at room temperature in the dark to detect the bound antibody. After extensive washing, the cells were analyzed immediately using a FACSCalibur system (BD Pharmingen, San Jose, CA, USA). Data were acquired and analyzed with the CellQuest software. Cells stained with sera from healthy subjects were designated as negative controls, whereas cells stained with sera from high-titer AChR patients (detected using the ELISA kit) were used as positive controls.

## Results

### Cell-based detection of antibodies to clustered AChRs in SNMG sera with confocal imaging

**Figure 1:**
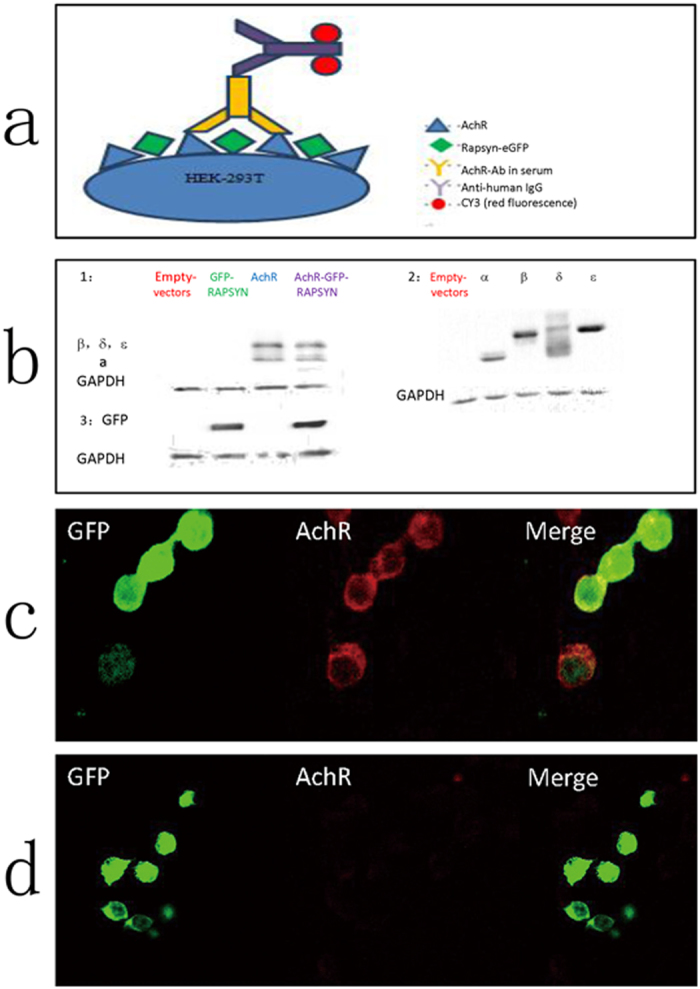
*Transfection of AChR subunits and confocal images of sera from SNMG patients.* (**a**) Diagram illustrating the principle of cell-based detection. (**b**) Transfected genes were verified by Western blotting, cropped blots are used in the figure, and the full figure is available in the supplementary information. HEK293T cells were divided into four groups: transfected with empty vectors as negative controls; transfected with vectors encoding GFP-RAPSYN only; transfected with four subunits of AChR without GFP-RAPSYN; and transfected with all five vectors (four subunits of AChR with GFP-RAPSYN). Expression of AChR subunits is shown in b1 and b2, and expression of GFP is shown in b3. (**c**, **d**) Samples of sera from an SNMG patient (c; positive control) and a healthy subject (d; negative control) were tested with the cell-based assay. Immunofluorescence co-localization of GFP and anti-AChR antibodies (red) was only observed in sera from the SNMG patients but not the healthy subjects. Magnification: ×400. Double-stained cells were counted by FACS for the statistical analysis shown in the next figure.

We transfected HEK293T cells with plasmids encoding different AChR subunits and rapsyn-GFP. The transfection efficiency for the AChR subunits was verified by western blotting, which exhibited clear bands corresponding to these subunits, whereas no bands were observed in the HEK293T control cells transfected with empty vectors or in the cells transfected with rapsyn-GFP only. In addition, GFP expression was only detected in the cells transfected with rapsyn-GFP or AChR-rapsyn-GFP (Fig. 1b). The transfection rate was further confirmed by flow cytometry, which exhibited >70% of GFP+ cells among GFP-transfected cells. These results confirmed that HEK293T cells had been successfully transfected with our target genes.

We then performed ELISAs to detect autoantibodies in the sera from MG patients. Among the 52 enrolled patients, 24 were classified as having SNMG. Thus, we next tested for serum anti-AChR antibodies in these patients using immunostaining of transfected HEK293T cells and confocal microscopy. As shown in Fig. 1d, transfected cells added to sera from SNMG patients exhibited both GFP expression and anti-AChR antibodies (red), which were bound to AChRs clustered on the surface of HEK293T cells. In contrast, cells added to sera from healthy subjects (negative control) showed only GFP expression, and the absence of anti-AChR antibodies. To avoid inaccurate results, we did not count double staining on the slides with the naked eye but instead performed a statistical analysis of the positive cells by flow cytometry (see below). Nonetheless, the results from confocal microscopy provided strong evidence for the feasibility of this cell-based detection of anti-AChR antibodies in SNMG patients.

### Flow cytometry

**Figure 2:**
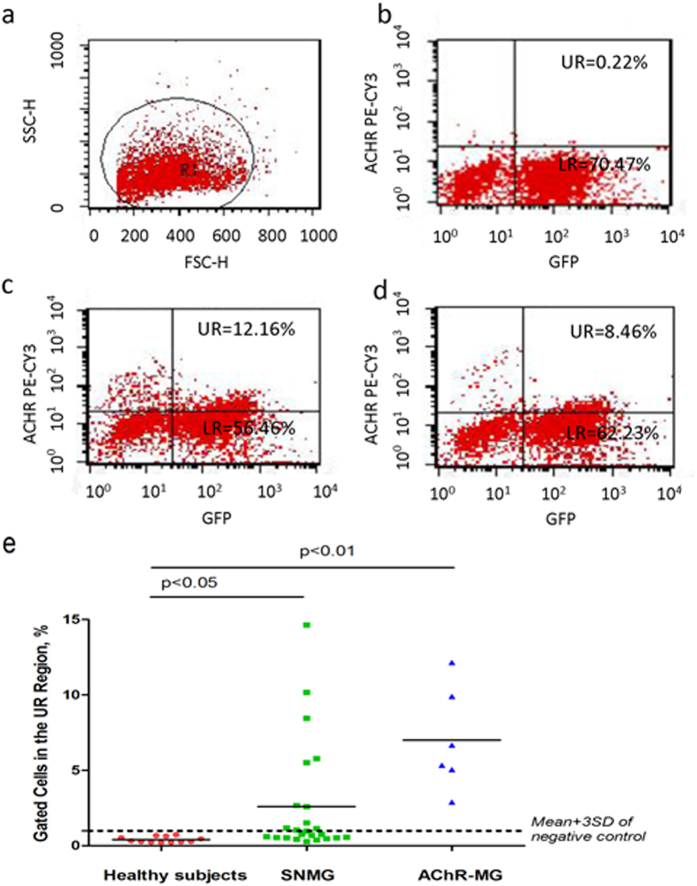
*FACS analysis.* Sera from different groups were incubated with transfected HEK293T cells and were assayed by flow cytometry. Cells gated in the upper right (UR) portion of the scatter plot represent double-stained cells, which were considered to be AChR-antibody positive. The cut-off was defined as the mean +3 SDs (=0.98%) of results from 12 healthy controls binding to clustered AChR. (**a**) Demonstration of the gating. (**b**) Scatter plots from FACS analysis of the negative control (healthy subject). (**c**) Scatter plots of a sample from an MG patient, whose serum was anti-AChR positive based on ELISA (AChR-MG), serving as the positive control. (**d**) A serum sample of an SNMG patient. (**e**) Summary of FACS analysis results of the three groups. n = 12 in healthy subjects (negative controls), n = 24 in SNMG patients, and n = 6 in AChR-MG patients (positive controls).

We then performed flow cytometry to confirm the immunostaining results of the transfected cells after incubation with different groups of sera. Among all of the gated live cells (Fig. 2a), green fluorescence represented cells transfected with GFP-rapsyn, which showed a transfection efficiency of >70% (Fig. 2b–d; gated cells in the right portion of the scatter diagram). The cut-off for positive cells was defined as the mean +3 standard deviations (SDs) (0.98%) of the results from 12 healthy controls (statistical analyses were performed with the SPSS software, version 18.0), and the cells in the upper right region (double positive) represented transfected cells bound to serum anti-AChR antibodies. As shown in Fig. 2b, there were very few AChR positive cells among the transfected cells incubated with sera from healthy subjects (negative control), whereas the sera from AChR-MG patients (positive control) contained AChR-positive cells (Fig. 2c). Importantly, 11 out of 24 SNMG (45.83%) cases were AChR-Ab positive using this method (Fig. 2d). There was a significant difference in IgG binding between the sera from SNMG and AChR-MG patients and sera from healthy subjects (P < 0.05–0.001; Fig. 2e).

### Clinical summaries of 11 cases of SNMG

Patients without anti-AChR or MuSK antibodies were referred to as SNMG patients. We retrospectively studied the data from 24 SNMG patients who, based on both primary and supplementary criteria, were diagnosed with MG at the First Affiliated Hospital of Second Military Medical University. Patients with antibodies to clustered AChRs were found to account for 45.83% (11 out of 24) of our SNMG patients, and the clinical characteristics of these patients were analyzed further (Table 1). Of these patients, there were 7 men and 4 were women, with the age of disease onset ranging from 19 to 63 years old (mean: 41 years). Ocular weakness was the most common symptom of MG, and there was a high incidence of bulbar weakness with painless dysphagia, dysarthria, or chewing difficulties. The patients were in different stages of the disease course, with maximal severity ranging from Osserman classification II to III^14^. Patients 4, 6 and 10 were at the peak of disease severity, whereas patients 1, 5 and 9 had already greatly improved following treatment. Five patients had early onset of MG (prior to 40 years of age), and five were thymectomized. These SNMG patients shared a similar clinical presentations as the AChR antibody-positive MG patients.

## Discussion

The present study proved that a novel cell-based assay could improve the detection rate of AChR-antibody-positive MG patients, particularly SNMG patients. Previous studies have shown that SNMG was similar to AChR-MG in terms of disease pathophysiology, symptoms associated with weakness, and the distribution of treatment responsiveness^12^. We therefore hypothesized that anti-AChR antibodies were generated in some of the SNMG patients but could not be identified by routine assays. There are several possible explanations for the failure to detect AChR antibodies in SNMG patients. While antibodies to another NMJ protein might exist, the most plausible explanation is the failure of current methods to detect the antibodies, given the greater similarity in the clinical features of SNMG and AChR-MG patients. Specifically, the antigenic determinants could be lacking in the solubilized AChR used in the traditional assay, or the failure might occurred due to the low affinity of AChR antibodies for soluble AChRs. Without clustering, the metabolic stability of AChRs decreases, resulting in false negatives^15^. Our findings demonstrated convincingly that there was an immune response to the AChR when the antibodies clustered with rapsyn in some of the patients whose serum antibodies were not detectable with routine immunoprecipitation assays. A further difficulty in diagnosing was due to some patients already being on immunosuppressive treatment and a few of them having even been thymectomized before sampling. These interventions could have affected the efficacy of detecting different antibodies.

Rapsyn was of great importance for the construction of the clustered AChRs model^16^. Indeed, our study showed that, when rapsyn-clustered AChR was expressed in its native conformation at a density similar to that at the NMJ, AChR antibodies could be detected in 45.83% (11/24) of serum samples that were previously negative for binding to AChR in solution. The positive cells were confirmed by FACS analysis to be double-stained cells, and we considered some of them to be negative results when they did not reach the standard that we had set, 0.98%, which was obtained from the sera from 12 healthy subjects (data shown in Fig. 2b–e). Given the inaccuracy and difficulty of counting double-stained cells with the naked eye using a microscope, we used FACS analysis to count these cells, and we believe this method to be objective, accurate and sufficiently powerful to replace the time-consuming and subjective manual counts.

Our results have important clinical and technical implications because they could eventually provide the basis for a new assay for the detection of AChR and other antibodies in MG, and approaches such as those that we used could become part of routine practice for diagnosis of the growing number autoantibody-mediated diseases of the peripheral and central nervous systems. Although there are other types of available assays, cell-based assays offer specific advantages. For instance, the antigen is expressed on the surface of HET293 cells, and only extracellular epitopes will be detected if the cells are alive and unpermeabilized. In contrast, proteins used in other assays, such as ELISA, are usually recombinant and are produced in bacterial expression systems, with the potential of being incorrectly folded. In addition, antibodies to intracellular epitopes are often detected, which are unlikely to be pathogenic. Another advantage of cell-based assays is that high expression levels can be guaranteed on the cell surface by including transient-transfection of a clustering protein, thus improving binding sensitivity. In contrast, rapsyn-induced clustering could not be achieved in the process of artificial antigen coating^17^. In animal experiments, the pathogenicity of anti-AChR-antibodies could be determined according to rapsyn expression, whereas declustering of AChRs could be associated with reduced antibody binding^18^. These results raised the possibility that changes in the packing geometry of clusters or intracellular modifications of the AChR might influence the binding of low affinity antibodies^19^.

In summary, our results demonstrated that AChR antibodies could be detected in approximately half of the so-called SNMG patients, using our cell-based assay. This technique represents a more sensitive approach than classic ELISA, thus increasing the diagnosis rate and reducing the frequency of SNMG^20^. This study was the first time that cell-based detection of MG was applied in the Chinese population. Furthermore, this approach avoided any injury to the medical staff due to radioactive materials. Thus, using simple and accurate flow cytometry, cell-based assays have the potential of being incorporated into routine diagnosis and management, to increase the sensitivity and specificity (as shown in our study) of autoantibody detection in SNMG.

## Additional Information

**How to cite this article**: Zhao, G. *et al*. Clinical application of clustered-AChR for the detection of SNMG. *Sci. Rep.* **5**, 10193; doi: 10.1038/srep10193 (2015).

## Supplementary Material

Supplementary Information

## Figures and Tables

**Table 1:** 

**Patient**	**Sex**	**Osserman classification**	**Age of onset**	**Ptosis**	**Weakness of limbs**	**Thymoma**	**Dyspnea**	**Bulbar weakness**	**Treatment**
1	M	I	58	1	0	0	0	0	A
2	M	IIb	36	0	1	1	0	1	A+CS
3	M	IIa	63	1	1	0	0	0	A
4	F	III	23	1	1	0	1	1	A+AZA+CS+PE
5	F	I	32	1	0	1	0	0	A
6	M	III	44	0	1	0	1	1	A+CS+Ig
7	F	IIb	19	1	1	1	0	1	A+AZA+CS
8	M	IIb	20	1	1	1	0	1	A+AZA+CS
9	M	I	56	1	0	1	0	0	A
10	F	III	43	1	1	0	1	1	A+CS+Ig
11	M	IIa	53	1	1	0	0	1	A+CS

*M, male; F, female; A, acetylcholinesterase inhibitors; AZA, azathioprine; CS, corticosteroids; Ig, polyclonal immunoglobulin; PE, plasma exchange.*
