# Supplementary material for: Clinical application of clustered-AChR for the detection of SNMG
Source: Sci Rep. 2015 Jun 11;5:10193. doi: 10.1038/srep10193 (PMC4464178; doi:10.1038/srep10193)
Supplement: Supplementary Information [file srep10193-s1.pdf]

Clinical application of clustered-AChR for the detection of SNMG

Guang Zhao   Xiaoqing Wang   Xiaowen Yu   Xiutian Zhang

Jianming Jiang*   Yangtai Guan [#]

1: Western blot :

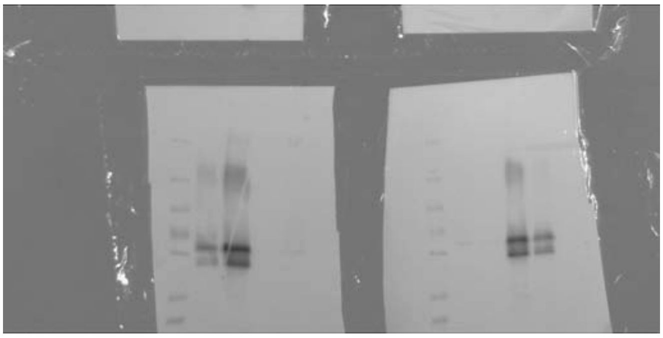

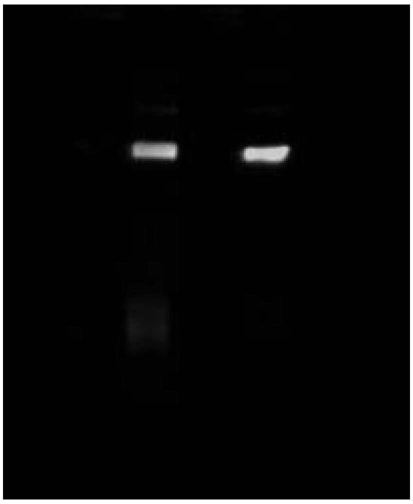

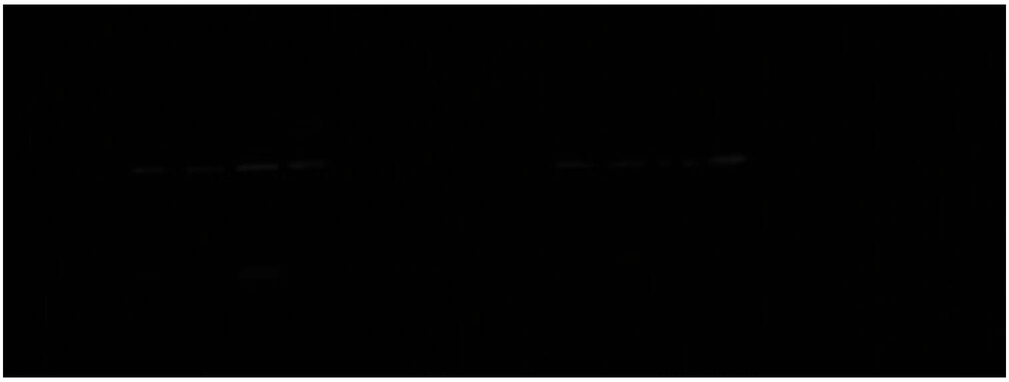

2: Western blot :

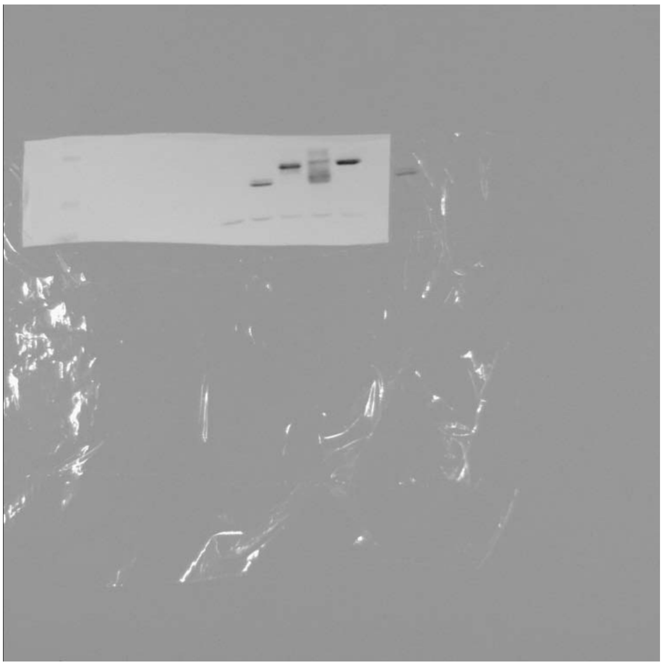

3. Double positive cells percentages (using Flow cytometry)

Healthy subjects	SNMG	AChR-MG (unit:%)
0.32	5.52	12.16
0.73	5.79	6.68
0.53	1.05	5.34
0.28	10.19	5.05
0.64	8.46	9.91
0.45	14.65	2.89
0.21	1.15	
0.25	1.53	
0.23	2.68	
0.27	2.59	
0.22	1.18	
0.67	0.55	
	0.70	
	0.61	
	0.38	
	0.43	
	0.97	
	0.54	
	0.77	
	0.48	
	0.29	
	0.76	

0.53
0.58
